# Self-managing symptoms of Long COVID: an education and strategies research protocol

**DOI:** 10.3389/fpubh.2024.1106578

**Published:** 2024-02-07

**Authors:** Julia Rybkina, Nithin Jacob, Brenda Colella, David Gold, Donna E. Stewart, Lesley A. Ruttan, Liesel-Ann C. Meusel, Mary P. McAndrews, Susan Abbey, Robin Green

**Affiliations:** ^1^KITE Research Institute, Toronto Rehabilitation Institute—University Health Network, Toronto, ON, Canada; ^2^Telerehab Centre for Acquired Brain Injury, Toronto Rehabilitation Institute—University Centre, University Health Network, Toronto, ON, Canada; ^3^Krembil Brain Institute, University of Toronto, Toronto Western Hospital, University Health Network, Toronto, ON, Canada; ^4^University of Toronto, Centre for Mental Health and Senior Scientist, University Health Network, Toronto, ON, Canada; ^5^University of Toronto Scarborough, Neuro-Rehab Program, Toronto Rehabilitation Institute—University Centre, University Health Network, Toronto, ON, Canada; ^6^Krembil Research Institute, University of Toronto, University Health Network, Toronto, ON, Canada; ^7^Medical Psychiatry and Psychiatry and Psychosocial Oncology, University Health Network, Toronto, ON, Canada; ^8^Department of Psychiatry, Division of Neurosciences and Clinical Translation, University of Toronto, Toronto, ON, Canada

**Keywords:** Long COVID, COVID-19, rehabilitation, intervention, self-management

## Abstract

Post-acute sequelae of SARS-COV-2 (PASC) is growing in prevalence, and involves symptoms originating from the central neurological, cardiovascular, respiratory, gastrointestinal, autonomic nervous, or immune systems. There are non-specific symptoms such as fatigue, headaches, and brain fog, which cannot be ascribed to a single system. PASC places a notable strain on our healthcare system, which is already laden with a large number of acute-COVID-19 patients. Furthermore, it impedes social, academic and vocational functioning, and impacts family life, relationships, and work/financial life. The treatment for PASC needs to target this non-specific etiology and wide-ranging sequelae. In conditions similar to PASC, such as “chemo brain,” and prolonged symptoms of concussion, the non-specific symptoms have shown to be effectively managed through *education and strategies for self-management* and *Mindfulness* interventions. However, such interventions have yet to be empirically evaluated in PASC to our knowledge. In response to this gap, we have developed a virtual education intervention synthesized by psychiatrists and clinical psychologists for the current study. We will undertake a two-phase randomized controlled trial to determine the feasibility (Phase 1; *N* = 90) and efficacy (Phase 2; sample sized based on phase 1 results) of the novel 8 week Education and Self-Management Strategies group compared to a mindfulness skills program, both delivered virtually. Main outcomes include confidence/ability to self-manage symptoms, quality of life, and healthcare utilization. This study stands to mitigate the deleterious intrusiveness of symptoms on everyday life in patients with PASC, and may also help to reduce the impact of PASC on the healthcare system.

**Clinical trial registration:**https://classic.clinicaltrials.gov/ct2/show/NCT05268523; identifier NCT05268523.

## Introduction

### Long-COVID: diagnosis and epidemiology

The coronavirus disease 2019 (COVID-19) is caused by the severe acute respiratory syndrome coronavirus 2 (SARS-CoV-2) (1). While the word “acute” implies a brief illness (2), many patients experience persisting symptoms (3). These ongoing and sometimes debilitating symptoms were initially brought to public attention by online social support groups that quickly proliferated early in the pandemic (4); these groups coined the terms “Long-Hauler and ‘Long-COVID.” Since then, prolonged symptoms have been additionally referred to as *post-COVID syndrome*, *long-haul COVID-19*, and *post-acute sequelae of SARS-COV-2* (PASC) (4). The labels initially referred to patients with symptoms lasting more than one month following diagnosis of SARS-CoV-2 (5–8), and now typically refer to symptoms lasting three or more months (9–11). Here, we use the term PASC because it is explicit in its focus on long-lasting symptoms of COVID-19 without etiological assumptions of symptoms.

Like the acute illness, enduring symptoms of COVID-19 are often multi-faceted (12–15). Origins of symptoms may be central neurological (e.g., cognitive impairment, depression/anxiety, loss of sense of smell/taste) (14, 16), cardiovascular (e.g., chest pain/tightness, tachycardia) (17, 18), respiratory (e.g., dyspnea, cough) (14, 19–23), gastrointestinal (e.g., nausea, abdominal pain, altered gut microbiota) (24–26) and/or musculoskeletal (e.g., joint/muscle pain) (27). Symptoms may also have their origins in the autonomic nervous (28) or immune systems (inflammatory and neuroinflammatory) (29). While some symptoms can be ascribed to specific etiology, there are also symptoms where the pathophysiology and causes are uncertain (30).

The exact prevalence of PASC remains uncertain, but given the vast numbers of patients sustaining SARS-CoV-2 – nearly 500 million at the time of writing (31) – even the smallest estimates are concerning, especially as protracted sequelae occur even in mild acute illness and in young and fit patients. One of the largest meta-analyzes to date, pooling 54 studies and almost 1.2 million patients’ data from around the world, found that 6.2% of patients experienced symptoms lasting beyond 4 weeks (32). Other smaller-scale studies have reported prevalence rates varying between 10 and 65% (33–35).

Not surprisingly, the societal impact of PASC is significant. For instance, SARS-CoV-2 is estimated to share a similar mortality rate to that of seasonal influenza, causing an estimated doubling proportion of deaths annually (36). Moreover, the increased healthcare utilization places a notable strain on a system already burdened by large numbers of patients and healthcare workers with acute COVID-19 (28, 37). A retrospective cohort study in Canada found that the mean days in hospital per-person-year increased by 47–53%, 8 weeks or more after a COVID-19 infection (38). Moreover, the array of cognitive, physical and emotional sequelae of PASC impede social, academic and vocational functioning with deleterious financial consequences (39). A large-scale survey of >3,000 patients with PASC (40) found 71% of respondents endorsing compromise to family life and relationships, with 31% endorsing reduced ability to care for children and dependents. Work-related impacts were endorsed by 81% of people, and 36% endorsed financial impact. Similarly, Davis and colleagues (14) in a survey of 3,762 participants with PASC found that 45% needed a reduced work schedule, and 22% were not working due to illness at 6 months post-diagnosis.

Given the above consequences, a treatment for PASC is needed to reduce its burden on patients and society. However, the wide-ranging non-specific and specific nature of symptoms has made this a significant treatment challenge (41).

### Identifying treatment options: overlap of PASC with related disorders

One path for treating PASC is to identify other disorders with an overlapping clinical presentation that have treatments with demonstrated efficacy (42). PASC shares a likeness with several conditions in which a discrete organic event is followed by a cluster of prolonged and non-specific symptoms. These include “chemo brain” (43–47), post-viral syndromes such as post influenza, SARS (48), Middle East Respiratory Syndrome (49), myalgic encephalomyelitis/chronic fatigue syndromes (50), and the prolonged symptoms of concussion (51). In all of these disorders, symptoms can include fatigue, sleep disturbance, brain fog, headache and mood disturbance, for example, which are some of the more common non-specific PASC sequelae.

Of particular relevance to PASC is the idea of symptom interplay and exacerbation. This has been described in the concussion literature from a “network perspective,” whereby non-specific symptoms amplify and reinforce one another (52). For example, fatigue may cause or exacerbate cognitive symptoms, which may cause or escalate mood symptoms, which may in turn worsen sleep (53).

The diagnostic challenges of non-specific and trans-diagnostic symptoms in post-viral syndromes, chemo brain and prolonged symptoms of concussion have led to treatment approaches that do not necessitate a mapping between symptom and cause at the individual patient level. One such approach is *education and strategies for symptom self-management* (e.g., educating about the disorder and expectations regarding symptom recovery; providing strategies for managing one’s own symptoms), which has shown efficacy for managing non-specific and specific symptoms associated with post-viral syndromes, and chemo-brain (54). Education has also been found to be prophylactic against the development of prolonged symptoms of concussion (55). Not surprisingly, education and strategies have been advocated for managing prolonged COVID-19 symptoms (56), but remain to be empirically tested in this population. Another approach with efficacy for non-specific and specific symptoms is *Mindfulness* (referring to increasing one’s awareness of the present moment) (57), and which can enhance an individual’s internal locus of control for optimizing health (58). Mindfulness treatments have been shown to improve: fatigue in patients with cancer (59–61); anxiety, depression and quality of life in patients with myalgic encephalomyelitis/chronic fatigue syndromes (62); chronic pain, depression relapse, addiction (57); and, symptoms of fatigue and depression in concussion (54, 63). Mindfulness interventions have also shown to improve prolonged symptoms of concussion (63–65). In the context of PASC, however, Mindfulness has limitations for certain physical symptoms such as shortness of breath, lung function, or exercise intolerance (66).

### Limited research into current approaches to treatment of PASC

While a number of clinical recommendations have been made for PASC patients, there is a paucity of treatment research to date. The recommended pharmacological approach is symptom-based; this has mainly included the repurposing of drugs that are employed for similar conditions and symptoms (5, 67). For example, medications for chemotherapy and brain fog [e.g., methylphenidate, donepezil, modafinil, and memantine (68)] are under consideration for brain fog in PASC (69). However, currently, no drugs have been found to effectively target subjective cognitive symptoms of PASC, and studies investigating their efficacy are still limited to case-studies (70).

A number of recommendations have been made to promote self-education and strategy use. The National Institute for Health and Care Excellence (NICE) guidelines recommend directing patients to online resources and apps to optimize self-management, for example referring to behavioral guidelines published by the WHO (71) and a PASC self-management web-page created by the Provincial Health Services Authority in Canada (72). As well, a recent guide published for primary care physicians advocated educating patients with PASC with concrete strategies and recommendations for managing their respiratory, cognitive, neurological, and somatic symptoms (73). To date, there are no published multidisciplinary education, strategy and self-management approaches for PASC.

### Aims and hypotheses

In sum, while management of acute COVID is being studied extensively, there is no candidate treatment that targets, let alone alleviates, all or most symptoms of PASC across patients. In response to this gap in care, we developed an education and strategies intervention inspired by findings in related populations that have shown efficacy. This intervention synthesized by psychiatrists and clinical psychologists provides practical recommendations to manage both the specific and non-specific symptoms of PASC. The current study aims to (i) demonstrate the feasibility and efficacy of a novel education and strategies intervention for self-management of PASC symptoms that can be delivered cost-effectively, (ii) and to disseminate an intervention package to aid symptom self-management free-of-charge to licensed therapists for delivery to their patients with PASC. The intervention package will be fully manualized, with (i) videos of presentations from clinician-scientists (cardiology, neurology, respirology, rheumatology, psychology) with experience in PASC that includes frequently asked questions from patients and answers from clinicians; and (ii) PowerPoint presentations plus scripts to be delivered by the facilitator.

The treatment under development employs remote delivery, which has shown to be cost-effective (74, 75), and can tackle the scale and geographic distance of patients from treatment centers. Remote delivery also prevents infection risk, employing a group-based approach which has shown to be cost-effective (76), and may be psychologically optimal given the proliferation of online groups for individuals with PASC, suggesting a need for mutual support. Importantly, group-based approaches for related disorders have been proven effective and feasible (77) as have telerehabilitation and education approaches (78).

We will undertake a two-phase randomized controlled trial (RCT) to determine feasibility and effect size (Phase 1) and efficacy (Phase 2) for a novel 8-week Education and Self-Management Strategies group compared to a mindfulness skills program. Phase 2 is a full-scale quantitative-only RCT, with refinements and power analysis based on Phase 1 results, and an additional third no-treatment control arm. Outcomes of interest include confidence and ability to self-manage symptoms, quality of life, healthcare utilization. Hypotheses: Both interventions will be associated with enhanced mood, anxiety, quality of life and management of dyspnea symptoms. The education and self-management strategies program will additionally increase confidence to self-manage symptoms, decrease intrusiveness of Long-COVID symptoms in everyday life, and decrease medical visits, more so than the mindfulness group.

## Methods

### Participants

Individuals (*N* = 90; >18 years) with previously diagnosed COVID-19 and experiencing symptoms lasting for more than 3 months (i.e., persisting symptoms) are currently being recruited for Phase 1. Sample size is based on paired tests from our prior concussion study (79) (*N* = 61; under review) that used the same primary outcome. With effect size 0.59 and standard alpha and beta values, 26 pairs are needed. Factoring (i) 20% attrition (conservative given 7% attrition in a similar concussion study [79; under review] and 9% in the pilot group for the present study), and (ii) a further 25% for 1-month follow-up, we will recruit N = 45 for education of self-management strategies group and N = 45 for mindfulness skills group. Key inclusion criteria are: positive COVID-19 diagnosis a minimum of 3 months prior to study participation; proof of COVID-19 diagnosis through PCR, rapid antigen or serology test; self-report experiencing 3 or more persisting symptoms from 2 or more categories of mood, cognitive and somatic domains; age > 18; and English speaking. Key exclusion criteria include past acute ventilator support; past or present major neurological/psychiatric conditions including dementia, mild cognitive impairment, psychotic illness, mania, chronic fatigue syndrome, fibromyalgia, chronic Lyme disease, and/or traumatic brain injury.

### Study design

**Figure 1:**
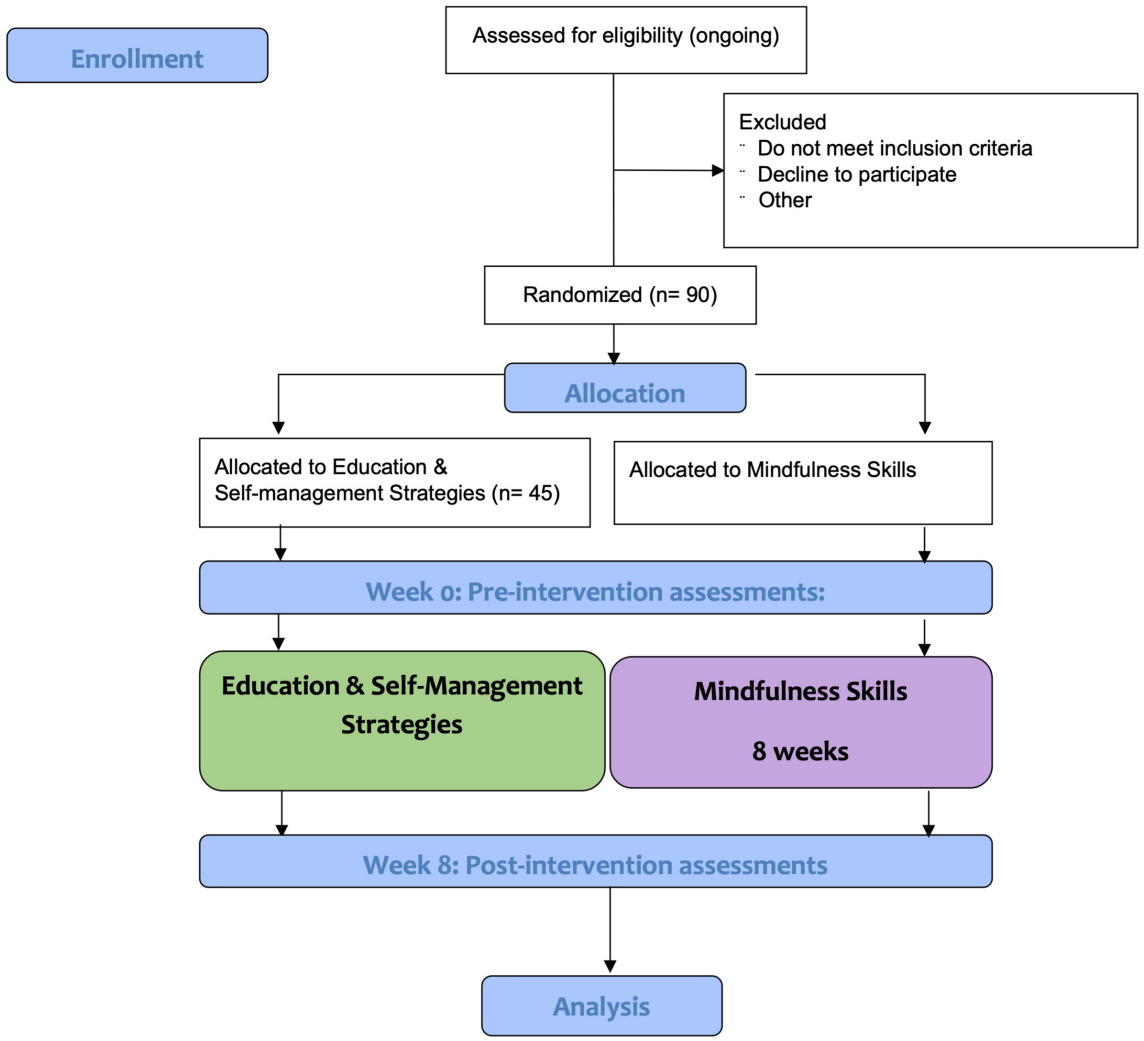
CONSORT study flow diagram of the present protocol; CONSORT, Consolidated Standards of Reporting.

The Phase 1 trial is a block randomized, controlled, parallel group, two arm, assessor-blinded trial with a 1:1 allocation. Participants will be randomly assigned to either the Education and Self-Management Strategies or the Mindfulness Skills groups. The allocation sequence is determined using the random function in Microsoft Excel by the study coordinators prior to recruitment. The study coordinators will screen, consent, enroll, and assign participants to their study identity numbers. Qualitative assessment of *N* = 20 patients from the Education and Self-Management group will be purposively (i.e., subjectively) sampled to attain a heterogeneous subset of patients. Qualitative information will be combined with quantitative to permit a more thorough understanding of the outcomes and to aid in further program refinement before undertaking the Phase 2 full-scale RCT. This study design is summarized in COSORT flow diagram presented in Figure 1.

Phase 2 will be a full-scaled trial with an additional third arm (1:1:1 allocation) as a non-treatment, standard-of-care group, with refinements and power analysis based on Phase 1 results.

### Procedures

For both groups, 8 weekly sessions will be delivered remotely on Microsoft Teams by licensed therapists (e.g., Psychologist, Occupational Therapist) with mental health experience; discipline experts will also deliver content for the Education and Self Management Strategies Group. Sessions will last 1.5 h, with 10–15 patients per group (factoring in attrition).

#### Education and Self-Management group protocol

All 8 sessions include education and independently deployable practical strategies (e.g., energy conservation, mindfulness and Cognitive Behavioral Therapy) and principles of self-compassion; self-care, cued diaphragmatic breathing, and phased return to physical activity that will be practiced during and between sessions. While the literature reveals that PASC symptoms can have specific aetiologies, the diagnosis of the etiology of a given symptom in a given patient may be uncertain. Moreover, the presence of some PASC symptoms may cause or exacerbate other symptoms (e.g., fatigue may result in or worsen attentional difficulties). Therefore, the education and strategies we provide are intended to be useful whether or not we have certainty of the underlying symptom cause in any given patient. Restated, the focus is on providing education and strategies for ameliorating symptoms, without making any assumptions about the cause of symptoms in any individual patient.

Benefits of this type of protocol (i.e., strategies and education) include non-invasiveness and no financial cost to patients. This is important given growing concern that in the absence of treatment for the wide-ranging symptoms of PASC, pernicious treatments (80–83) may fill the void.

Each session will end with a question-and-answer period. The content presented in each week is outlined in Table 1. During weeks 3–6, medical experts will deliver a 20–25-min presentation on their respective area of expertise and its relationship to Long-COVID, and provide self-management strategies.

**Table 1:** Overview of Education and Self-Management Strategies group protocol content.

Week	Topic	Subject expert(s)
1	Overview & Introduction	Psychologist
2	Intro to self-management strategies: discuss the importance of self-care/self-management of symptoms in recovery (e.g., Goal Setting, Energy Conservation, Managing Fatigue, Activity Scheduling, Sleep and Exercise/Nutrition)	Psychologist
3	Respiratory symptoms: presentation on the topic of long-term respiratory symptoms associated with COVID, recommendation of at-home breathing strategies and exercises (e.g., diaphragmatic breathing, active cycle breathing), discussion and question and answer period	Respirologist & Physiotherapist
4	Cardiac symptoms: presentation on the topic of persistent post-COVID heart-related symptoms and disorders, recommendation of a safe and gradual approach to returning to physical activity, potential role for multidisciplinary rehabilitation, when to see a specialist, discussion and question and answer period	Cardiologist
5	Rheumatological symptoms: presentation on the long-term rheumatological symptoms after COVID, discussion of recommended pharmacological and non-pharmacological self-management strategies focusing on (i) pain and inflammation control, and (ii) maintenance of physical function/avoidance of long-term deformities, question and answer period	Rheumatologist
6	Neurological & Mental Health Symptoms: overview of Long-COVID neurological and mental health symptoms, discussion of biological and environmental factors that can impact symptoms, recommendation of self-management strategies (patient organizations, exercise, socialization, self-compassion), question and answer period	Neurologist & Psychologist
7	Cognitive symptoms: presentation of cognitive symptoms associated with Long COVID, discussion of self-management strategies such as checking the basics (e.g., sleep, diet, physical exercise), incorporating concepts of neuroplasticity and environmental enrichment in daily life, question and answer period	Psychologist
8	Wrap up and review of clinical recommendations, self-management and self-care strategies	Psychologist

This table describes the structure and main focus of each session in the 8 week Education and Self-Management Strategies intervention, which are led by psychologists and other medical experts.

#### Mindfulness skills group protocol

The Mindfulness Skills intervention is an 8-week program designed to provide an introduction to basic mindfulness skills. Each session begins with a brief breath focus practice followed by discussion of the participants’ experience from the previous week. Each session also includes some didactics on a new related mindfulness skill that is introduced and practiced, followed by a question and answer period. Topics covered include breath focus practices, modes of being: autopilot vs. awareness, body scan practice, cognitive distancing strategies, and building a sustainable mindfulness practice. The content covered each week is outlined in Table 2.

**Table 2:** Overview of mindfulness group protocol content.

Week	Topic	Subject expert
1	Overview & Introduction	Psychologist
2	Two modes of being: Autopilot vs. Awareness; Exercise – Two ways of knowing (from Mindful Way -think about your feet vs. directly sense your feet – to help people understand the difference); Breath Focus practice; Mindfulness of everyday activities discussion	Psychologist
3	Introduction to the Body Scan; Body Scan practice/discussion	Psychologist
4	Using mindfulness responsively – introduce/practice Short Breathing Space (STOP – notice); Compassionate Body Scan practice/discussion	Psychologist
5	Mindfulness of Breath, Body, Sounds, Thoughts, and Choiceless Awareness	Psychologist
6	Review of cognitive distancing strategies/practice; Meditation/discussion – Loving Kindness for Ourselves	Psychologist
7	Review of cognitive distancing strategies/practice; Meditation/discussion – working with difficulty (from Mindful Way)	Psychologist
8	Building a Sustainable Mindfulness Practice; Guest House Poem – discussion Meditation – Compassionate Friend – Neff/Germer	Psychologist

This table describes the structure and main focus of each session in the 8 week psychologist-led mindfulness program.

### Outcome measures

After providing informed consent to participate in the study and prior to commencing the intervention groups, participants will be asked to complete an online background questionnaire in order to assess potential moderators. This background questionnaire collects demographic information (e.g., age race, sex, gender, highest education, occupation), previous and current medical and psychiatric history, current symptoms and healthcare supports.

#### Feasibility measures (Education and Self-Management group only; Objective 1)

(i) Recruitment (percentage of eligible participants that consented); retention (percentage of consented participants that completed the study); and adherence rates (percentage of sessions attended and outcome measures completed)
(ii) Standard Session Feedback Form: this 5-item patient reported outcome measure (PROM) was created by our group, and uses a 5 point Likert scale to measure the utility of content/strategies, amount of content, barriers to application, and any changes/benefits noticed from applying the strategies.
(iii) Exit interview: a semi-structured, qualitative interview comprising open-ended questions followed by probes. The interview probes pros/cons of intervention design and impact of the intervention on health and symptom self-management. Probes are based on the Workgroup for Intervention Development and Evaluation Research (WIDER) recommendations regarding content, format, delivery, timing issues and personnel (84). The interview will also contain probes for impact on health, health-related actions, including self-management of Long-COVID symptoms and health care visits.

#### Efficacy measures (Objective 2)

All primary and secondary outcomes are PROMs. Table 3 summarizes the timeline of the outcome measure collection.

(i) Primary outcome:

- Perceived Medical Condition Self-Management Scale (85) (PMCSMS). An 8 item tool using a 6-point scale to measure confidence to self-manage symptoms, with strong validity and reliability (85, 86), and associated with high treatment adherence and beneficial outcomes (85).

(ii) Secondary outcomes:

- Utilization of Healthcare Services Questionnaire. This tool was created by our group to measure past and planned healthcare-related visits.
- Depression, Anxiety and Stress Scale (DASS-21 (87)). A 21-item tool that uses a 4-point scale to measure stress, depression and anxiety symptoms.
- Quality of Life Enjoyment and Satisfaction Questionnaire – Short Form (Q-LES-Q-SF (88)). A 16-time tool that uses a 5-point scale to measure quality of life and degree of enjoyment and satisfaction experienced by participants in various areas of daily living.
- Adapted Illness Intrusiveness Rating (AIIR (89, 90)). A 13-item scale using a 7-point scale to measure intrusiveness of symptoms in daily life.
- Life Orientation Test – Revised (LOT-R) (91). A 10-item tool using a 5-point scale to measure dispositional optimism.
- International Physical Activity Questionnaire (IPAC) (92). An 8-item tool to measure engagement in physical activity.

(iii) Control/Moderator variables

- Brief COPE (93). A 28-item tool using a 4-point scale to measure ways of coping with stress in life.
- Somatic Symptom Scale – 8 (SSS-8 (94)). An 8-item tool using a 5-point scale to measure of somatic symptom burden.
- Multi-dimensional Health-Related Locus of Control (MHLC (95)). An 18-item tool to measure the locus of control for health-related behaviors.

**Table 3:** Summary and timeline of outcome measure collection.

Timeline		Outcome category	Outcome measures
Pre-intervention	Week 0; prior to first session	Feasibility (Objective 1)	Recruitment rate
		Efficacy (Objective 2)	Perceived Medical Condition Self-Management Scale Utilization of Healthcare Services Questionnaire Depression, Anxiety and Stress Scale Quality of Life Enjoyment and Satisfaction Questionnaire – Short Form Adapted Illness Intrusiveness Rating Brief COPE and Life Orientation test Somatic Symptom Scale Multi-dimensional Health-Related Locus of Control International Physical Activity Questionnaire
Intervention	Weeks 1–8	Feasibility (Objective 1)	Retention/adherence rate, Session Feedback Form
Post-intervention	Week 8; last session	Feasibility (Objective 1)	Qualitative exit interview (Education intervention group only)
		Efficacy (Objective 2)	(all efficacy outcomes mentioned above)
1-month follow-up (phase 1 and phase 2)	Week 12	Efficacy (Objective 2)	(all efficacy outcomes mentioned above)
3-month follow-up (phase 2 only)	Week 20	Efficacy (Objective 2)	(all efficacy outcomes mentioned above)

This table describes all the outcome measures and their timeline for administration. COPE: Coping Orientation to Problems Experienced Inventory.

### Statistical procedures

#### Feasibility and qualitative analyzes

Descriptive statistics will be used to summarize feasibility outcomes. Qualitative data are included to permit a deeper understanding of quantitative findings in this new population. A research assistant supervised by study investigator will use content analysis and constant comparison to identify, expand or merge themes in transcripts of recorded interviews, and develop a codebook of themes and exemplar quotes. We will tabulate themes by sex and within groups by sampling characteristics. This will identify common views about Education and Self-Management Strategies group design and its impact, yielding insight on how to further refine the intervention. By virtual meeting, the Research Team will interpret findings, which we will use to refine intervention design prior to the ensuing Phase 2 RCT.

#### Efficacy analyzes

To assess pre-to post-treatment and follow-up changes for the primary outcome (PMCSMS), we will undertake repeated measures group (Education vs. Mindfulness) by time (pre-, post-, and follow-up-education/mindfulness/waitlist group). ANCOVAs controlling for acute COVID-19 severity and time post-diagnosis. Secondary outcomes will also be examined in the same manner.

Hierarchical regression analyzes will be performed to examine impact of potential moderator variables on pre-to-post treatment improvement on the PMCSMS. In the first block, the moderators will be age, acute COVID severity, and time-post diagnosis. Sex and gender will be included in the second block, and Brief COPE, AIIR and MHLC will be added in the final block. Analyzes of repeated measures data will be performed using the Mixed Models for Repeated Measures (MMRM) methodology (96). We will employ a comparison of bias-corrected covariance estimators for MMRM analysis in longitudinal data with dropouts in order to accommodate the possibility of missing assessments or of assessments at different times post-diagnoses and to provide an effective framework for hierarchical regression analyzes and further exploratory analyzes (96).

## Discussion

The overall aim of this research is to create and disseminate an intervention package for the self-management of PASC symptoms. This brief, cost-effective and scalable intervention can be available to any licensed therapist with mental health expertise and enable treatment of patients regardless of geographic location or mobility restrictions and will enable treatment without concern for infection risk to patients or therapist. The current RCT protocol will determine if the Education and Self-Management Strategies group offers better (or at least non-inferior) symptom management outcomes compared with the Mindfulness and waitlist groups. The inability to attribute many symptoms to a natural cause may be anxiety-provoking for patients, which emphasizes the importance of education to understand the range of possible non-specific and specific symptoms, and mindfulness for accepting symptoms non-judgementally/agnostically and destigmatizing discomfort by alleviating secondary maladaptive attributions.

The increasing strain of PASC on healthcare resources may deleteriously affect the management and care of other patient populations (38, 97). Literature on the challenges of self-managing symptoms of Long COVID is limited at this time, however we can learn from research on other chronic conditions (e.g., chronic obstructive pulmonary disease, rheumatic diseases, kidney disease) that likely present similar challenges and limitations that individuals may encounter in their recovery journey (80, 98–100). Firstly, the heterogeneity of symptoms and their unpredictable nature make it challenging to develop universally effective strategies. What works for one person may not necessarily benefit another due to the diverse manifestations of Long COVID. Additionally, the limited understanding of the underlying mechanisms and long-term effects poses a challenge in tailoring targeted interventions. The fluctuating and persistent nature of symptoms can also create difficulties in establishing a consistent and manageable routine. Furthermore, the emotional toll of prolonged illness, including anxiety and depression, can impact one’s ability to effectively self-manage (98, 99). Access to healthcare resources and professional guidance is another limitation, as not all individuals may have equal opportunities to consult with specialists or access tailored medical advice (98–100). The proposed intervention, which provides accessible, remotely delivered education and teaching strategies pertaining to the diverse symptom domains of Long COVID, was designed to mitigate some of these challenges.

Hence, the study stands to reduce the suffering and improve the quality of life of patients with PASC. Recognizing the unique and diverse nature of symptoms, the strategies and education intervention protocol incorporates a combination of educational modules, teaching of practical strategies, interactive question and answer period and peer support, to empower participants with knowledge, skills, and a robust support system. Educational presentations are tailored to provide insights into the varied symptoms and their management, fostering a proactive approach to healthcare. A question-and-answer period ensures that individuals receive guidance specific to their experiences, promoting a targeted and effective self-management strategy. Peer support, on the other hand, creates a community where shared experiences foster mutual understanding and encouragement. The ultimate goal is to enhance the overall quality of life for participants by mitigating symptom burden and intrusiveness, improving coping mechanisms, and fostering confidence to self-manage. We anticipate that a well-rounded approach to self-management will not only alleviate symptoms but also positively impact mental health, thereby contributing to an improved quality of life. Moreover, the intervention may also help to reduce the impact of PASC on the healthcare system by empowering participants to manage their condition independently and helping patients to discern when to seek out physician support for symptoms and when to self-manage. This, in turn, may alleviate the burden on healthcare resources while offering individuals a sense of control and agency over their health. Through these interventions, our study aspires to bridge existing gaps in Long COVID care and contribute meaningful insights to the broader field of chronic illness management.

## Ethics and dissemination

This trial has already received ethics approval. If preliminary efficacy is observed in Phase 1 of the study, we will conduct a larger scale RCT at the University Health Network (UHN) in which we will provide clinical care through study participation in research. Control arm patients will receive treatment after the control phase. For the larger scale study, subject experts will create presentations that can be delivered by non-experts. We will manualize the entire protocol (comprising a clinician guide, PowerPoint presentations, FAQs), which will be made available to licensed therapists with mental health expertise. For rapid dissemination, the current protocol will be published online early in the study. Feasibility and preliminary efficacy results from the pilot will be submitted for publication as soon as the current pilot is complete. Full-scale results of the planned larger-scale RCT will be published first on bioRxiv (for more rapid sharing of information) and subsequently in peer- reviewed journals. Vehicles for knowledge uptake include: (i) local (Telerehab Centre at UHN; COVID-19 rehab clinic at UHN; CANCOV); Sunnybrook Health Sciences Centre, (ii) study team networks within Canada and abroad (e.g., ECHO COVID; International Neuropsychological Society).

## Ethics statement

This trial received ethical approval from the University Health Network Research Ethics Board (case #21-5038.0), and written informed consent was provided by the participants for participation.

## Author contributions

JR and NJ were involved in composing and revising the manuscript. BC, RG, SA, MM, L-AM, LR, DS, and DG were the original developers of the protocol, providing expertise, critical input, and editing throughout the writing process. All authors contributed to the article and approved the submitted version.
